# Host–Microbiome Interactions in a Changing Sea: The Gill Microbiome of an Invasive Oyster under Drastic Temperature Changes

**DOI:** 10.3390/microorganisms12010197

**Published:** 2024-01-18

**Authors:** Yahala Rina Dor-Roterman, Yehuda Benayahu, Leah Reshef, Uri Gophna

**Affiliations:** 1 School of Zoology, Tel Aviv University, Tel Aviv 69978, Israel; yahalarina@gmail.com (Y.R.D.-R.); yehudab@tauex.tau.ac.il (Y.B.); 2 Shmunis School of Biomedicine and Cancer Research, Tel Aviv University, Tel Aviv 69978, Israel; leahfa@tauex.tau.ac.il

**Keywords:** *Endozoicomonas*, Lessepsian migration, Mediterranean Sea, microbiome, oyster microbiota, *holobiont*

## Abstract

The gill tissue of bivalve mollusks hosts rich symbiotic microbial communities that may contribute to host health. *Spondylus spinosus* is an invasive Lessepsian oyster in the Eastern Mediterranean Sea that has become highly abundant while constantly expanding its range northwestward. Using 16S rRNA gene amplicon sequencing, we examined how temperature affects *S. spinosus* oysters and their gill microbiota in a series of experiments: exposing them to the current annual seawater temperature range, to the colder temperature of the Western Mediterranean Sea, and to the elevated temperature as predicted under global warming scenarios. The bacterial genus *Endozoicomonas* dominated the communities of the *S. spinosus*, mainly upon exposure to winter-like (16 °C) temperatures. Exposure to the elevated seawater temperature resulted in a significant change in the bacterial communities, while the oysters maintained normal functioning, suggesting that the oyster may survive a seawater warming scenario. Exposure to 11 °C led to the health deterioration of the oysters, the emergence of opportunistic pathogens, such as *Arcobacter*, *Vibrio*, *Colwelliaceae*, and *Pseudoalteromonas*, and a decline in the relative abundance of *Endozoicomonas*, suggesting that *S. spinosus* might not survive Western Mediterranean Sea winters. Both the host and its gill bacteria are thus greatly affected by temperature, which could consequently restrict the range of expansion of this and other invasive oysters.

## 1. Introduction

Organisms in all ecosystems, including marine ones, are crucially affected by temperature. Consequently, global warming is considered one of the major environmental concerns, posing a threat to marine biodiversity, as well as contributing to the invasion of marine species, particularly the movement of tropical species into subtropical and temperate seas [1]. Invasive species affect indigenous communities and, therefore, are considered a serious threat to biodiversity [2]. The Mediterranean Sea is undergoing a “tropicalization” process in which seawater temperature is constantly rising [3], followed by an increase in the introduction of invasive species. A major driver of this process was the opening of the Suez Canal in 1869, which connected the Mediterranean and the Red Sea [4]. This has led to a continued massive unidirectional invasion of Red Sea organisms, known as the Lessepsian migration [4,5].

A prominent Lessepsian invader is the oyster *Spondylus spinosus* Schreibers, 1793, which was first documented in the Eastern Mediterranean in 1988 [6]. It has since expanded northwest to Lebanon, Cyprus, Turkey, and Greece [7,8,9,10,11]. *S. spinosus* forms dense bed-like aggregations along the shallow Israeli Mediterranean coast and has the potential, therefore, to upset the equilibrium of the indigenous communities. *S. spinosus* is consequently considered one of the worst invasive species in the Mediterranean Sea, and is rapidly expanding its abundance there [12].

The possession of suitable symbiotic microbiota is crucial for a species’ success, as they may provide vital functions, such as supplying their host with nutrients, assisting in food digestion, and protecting against pathogens [13,14,15]. The relatively rich microbiota in bivalve gills [14,15] are considered to be stable autochthonous communities integrated within the tissues, unlike the microbiota in other organs, which can be variable and allochthonous [15,16].

Symbiotic microbiota are likely to be critical for invasion success [15]. We have previously shown a high degree of conservation in *S. spinosus* gill microbiomes in the Eastern Mediterranean and the Red Sea, which is indicative of a co-invasion by the host and its symbionts [17], although significant seasonal differences in the bacterial communities were noted. One of the greatest impediments to the process of biological invasion is that of contending with temperature differences between the source region and the invaded one [18]. Bacterial growth is highly sensitive to temperature changes, and therefore, the microbiome may be more susceptible to a shift in this environmental factor than its host, thus determining the distributional range of an invasive holobiont.

The temperature in the indigenous region of *S. spinosus* may fluctuate annually between 21 and 28 °C [19]; however, along the Israeli Mediterranean coastline, this oyster encounters a much broader temperature range of 16–31 °C [17]. The microbiome is considered to contribute to invasion success, and its response to such temperature differences may thus affect further trajectories of the oyster expansion. Here, we experimentally tested how temperature affects the microbiota of an invasive oyster. Specifically, this study examined the exposure of *S. spinosus* to the colder temperatures prevailing in the Western Mediterranean Sea and to the warmer temperatures predicted in this sea should global warming continue [20].

## 2. Materials and Methods

### 2.1. Experiment Design

**Figure 1:**
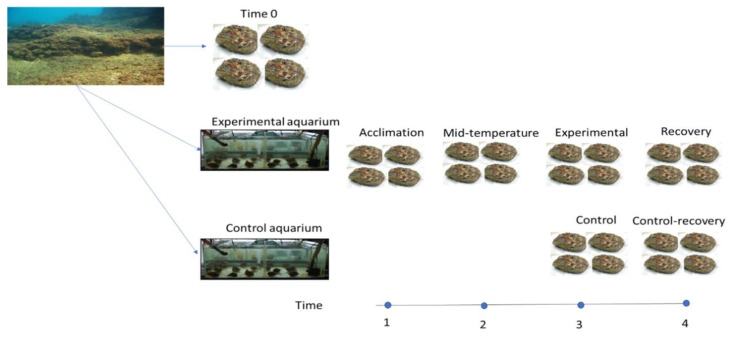
Schematic representation of experimental design; numbers indicate samples taken at different time points.

Temperature acclimation experiments were conducted over 1 year (Table 1A). In every experiment, oysters were removed with hammer and chisel from the substrate at Sdot-Yam site (32°29′26.0′ N 34°53′09.4′ E, 3–6 m depth) on the Israeli Mediterranean coast, placed into seawater buckets, and immediately transferred to the marine laboratory at Mevo’ot Yam, Mikhmoret. (Figure 1, Table 1). For each experiment, five oysters were randomly sacrificed for microbial analysis (procedure detailed below) and assigned to an inception group (time 0), thereby accounting for possible confounding effects of collection. The remaining oysters were divided into an experimental (300 L) and a control (150 L) aquaria. Each aquarium was supplied with a flow-through of 3 L/min while maintaining temperature, pH (8.3), and salinity (3.8%), corresponding to ambient Mediterranean conditions. Temperature was monitored four times a day (HOBO Pendant^®^ Temperature/Light 8K Data Logger, ONSET, Bourne, MA, USA), and ammonia, nitrite, nitrate, and phosphate levels were measured twice a week (API^®^ test kit, Livonia, MI, USA). Oysters were maintained under ambient temperature for one week of acclimation, after which four to five oysters were randomly retrieved and sacrificed for microbiome analysis to control for acclimation effects. The experimental aquarium was then exposed to a modified temperature change at a rate of 2 °C per day until reaching the desired temperature (Table 1A), while the control aquarium remained at ambient temperature. Following a two-week exposure to the target temperature, five to ten oysters from both experimental and control aquaria were sacrificed for microbiome analysis. The final phase was an adjustment of temperature in the experimental aquarium at a rate of 2 °C per day until returning to the ambient seawater temperature, at which point the oysters were sustained for an additional two weeks and then sacrificed for microbiome analysis as a recovery group. Concurrently, five oysters from the control aquarium were sacrificed for assessment as a ‘control-recovery’ group (allowing a comparison between the oysters that recovered from a temperature shift and ones that began the experiment at the same time but did not experience any temperature shift). The ‘extreme cold’ experiment (see above) lacked a recovery phase due to insufficient oyster supply.

Unless otherwise noted, all oysters demonstrated normal behavior, exhibiting immediate valve closing as a response to external stimuli, and soft tissues were intact and normally colored. The oysters were dissected under sterile conditions, which included surgically removing the gills, placing them on ice, and immediately transferring them to Tel Aviv University, where they were stored at −20 °C until further processing.

### 2.2. DNA Extraction, Library Preparation, and Deep Sequencing

All gill samples were homogenized using a 24-tube vortex apparatus at top speed for 10 min with glass beads, and total DNA was extracted using the PowerSoil DNA extraction kit (MoBio, Carlsbad, CA, USA), according to the manufacturer’s protocol, then stored at −20 °C. The V3–V4 region of the 16S rRNA gene was amplified using universal prokaryotic primers CS1-341F (5′-ACACTGACGACATGGTTCTACANNNNCCTACGGGAGGCAGCAG) and CS2-806R (5′-TACGGTAGCAGAGACTTGGTCTGGACTACHVGGGTWTCTAAT). Twenty-nine PCR cycles (95 °C for 15 s, 53 °C for 15 s, 72 °C for 16 s) were conducted, and successful amplification was verified using agarose gel electrophoresis. The PCR products were shipped to the Chicago Sequencing Center at the University of Illinois for paired-end deep sequencing on an Illumina MiSeq platform.

### 2.3. Microbiome Data Processing

Raw 16S rRNA gene amplicon data were processed using a custom pipeline incorporating scripts from the Quantitative Insights into Microbial Ecology v1.9 (QIIME) software package [21] and VSEARCH v2.15 package [22]. Demultiplexed raw sequences were quality filtered (removing bases with a PHRED quality score < 20), length filtered (discarding sequences < 380 bp), and merged using PEAR paired-end read merger. Data were then processed using a custom pipeline incorporating scripts from the Quantitative Insights into Microbial Ecology (QIIME) software package and VSEARCH package. Sequences were de-replicated and sorted prior to clustering at 99% identity; to reduce formation of spurious clusters, hereafter referred to as operational taxonomic units (OTUs), only sequences repeated more than five times (100% similarity) were allowed to form new OTUs. Chimeric OTUs (identified by uchime with the reference option ‘ref’, using the gold.fa database) were removed. OTUs were then assigned a taxonomy using the UCLUST [23] algorithm and Silva v128 database. Sample depth (post filtration) ranged from 3840 to 33,000 seqs/sample; to eliminate bias due to depth variation, data were rarefied to an even depth of 3840 seqs/sample.

### 2.4. Statistical Analysis

UniFrac-based distance matrices, obtained from QIIME, were exported to PAST, a statistical data analysis package [24], used to perform analysis of similarity (ANOSIM) [25] and principal coordinates analysis (PCoA). Permutational analysis of variance (PERMANOVA) was carried out in R using vegan v2.6 package. The LDA Effect Size (LEfSe) [26] biomarker discovery algorithm was applied to identify which bacterial taxa contribute to the differences between any two groups; *p*-value for the 1st (Kruskal–Wallis) step was set at 0.05, and LDA minimal threshold was set at 3.

## 3. Results

Three seasonal temperature-acclimation experiments were conducted throughout a single year, as detailed in the Materials and Methods section and summarized in Figure 1 and Table 1. Overall, 50 distinct bacterial phyla were represented in the gill tissues of *S. spinosus*. The phylum proteobacteria dominated, representing 67 ± 1.5% (*n* = 136, mean ± standard error) of the total microbiota. Within the proteobacteria, the bacterial communities were primarily composed of the classes γ-proteobacteria (45 ± 1.8%) and α-proteobacteria (12 ± 1%). *Endozoicomonas*, of the γ-proteobacteria class, was the most abundant genus (26 ± 2%) in the gill bacterial communities.

### 3.1. The Effect of Seasonal Temperature Patterns on the Gill Microbiome

**Figure 2:**
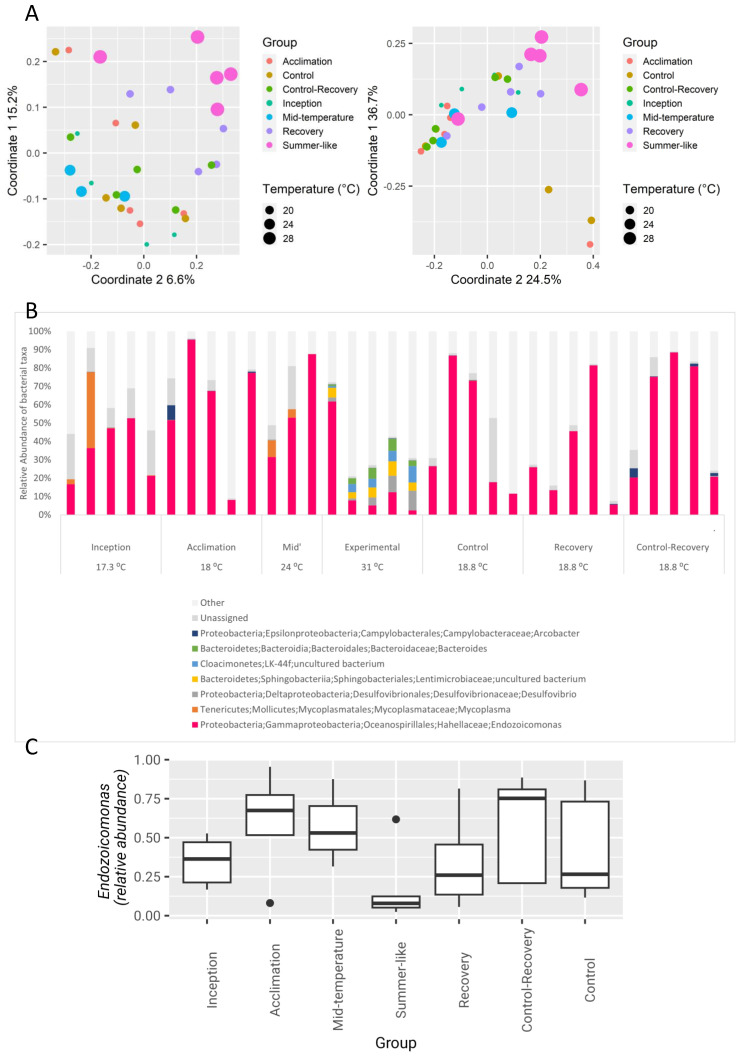
Effect of a winter-to-summer temperature shift on gill microbiome. (**A**) PCoA of weighted/unweighted unifrac matrices. (**B**) Relative abundances of dominant genera (appearing in at least 2 samples with a minimum RA of 5%). (**C**) *Endozoicomonas* relative abundance across all treatment groups.

Oysters were collected in winter (seawater temperature 17 °C) and exposed to a gradual temperature increase up to 31 °C (‘summer-like’), followed by a recovery period, during which the temperature was gradually reduced to the ambient temperature (19 °C, Table 1B); a control aquarium was kept at ambient temperature through the experiment. Principal coordinate analyses (PCoA) based on either weighted or unweighted UniFrac distances (Figure 2A) indicated a distinct separation of the summer-like group from the control ambient and mid-temperature groups. The recovery group samples tended to cluster in between the summer-like and ambient groups. PERMANOVA analysis detected significant effects on the microbial composition for temperature (*p* = 0.004, R = 0.11), as well as for the experimental group (*p* = 0.008, R = 0.23), and pair-wise ANOSIM (Analysis of Similarities) revealed a strong and significant separation between the summer-like group and all ambient groups pooled (*p* = 0.003, R = 0. 32; *p* = 0.001, R = 0.28, for weighted and unweighted UniFrac-based distances, respectively).

To further inspect microbial patterns driving the compositional shift of the summer-like group, the dominant genera per sample are shown in Figure 2B. Generally, communities of the summer-like group were more diverse than those of the ambient temperature ones (median Shannon diversity values of 4.21 vs. ≤3.12, respectively). Members of the Cloacimonetes phylum were detectable only in the summer-like group, reaching a relatively high median relative abundance of 5 ± 3%. Additionally, prevalent taxa in the summer-like group included members belonging to *Desulfovibrio* (median 5 ± 3%; class γ-proteobacteria), the family *Lentimicrobiaceae* (phylum Bacteroidetes, median 6 ± 2%), and the genus *Bacteroides* (median 4 ± 3%), all of which were barely detectable in all other groups (median 0%, max 0.3%; Kruskal–Wallis *p*-value for all comparisons between 0.009 and 0.0003; Supplementary Figure S1). Conversely, members of the order *Xanthomonadales* (class γ-proteobacteria) and the genus *Ruegeria* (class α-*proteobacteria*) were detectable in all groups but portrayed an increase in the summer-like group, which was retained during the recovery process (*p =* 0.008, *p* = 0.06, respectively; Supplementary Figure S1).

In agreement with a trend of increased diversity at summer-like temperature, an LDA effect size algorithm (LEfSe, Supplementary Figure S2), comparing the summer-like and ambient groups, identified 31 bacterial taxa that were overrepresented in the summer-like group, but only 6 that were overrepresented in the ambient group. A major determinant among the latter was the genus *Endozoicomonas*, demonstrating an LDA effect size of 4.5 for the separation between groups. In accordance with previous studies showing members of the genus *Endozoicomonas* to be more abundant during winter than during summer [17], *Endozoicomonas* levels decreased during summer-like conditions but returned to their initial levels in the recovery group (Figure 2C).

### 3.2. The Effect of Extreme Temperature Scenarios on the Gill Microbiome

Given the substantial effect on the gill microbiome composition as a result of a temperature increase, we next addressed the issue of microbiome modification at higher-than-normal temperatures. Oysters in the experimental aquarium were exposed to two extreme temperature conditions (32 and 33 degrees; ‘Extreme-first’ and ‘Extreme-second’, respectively). The control aquarium was sampled concurrently with both extreme temperature sampling groups, thus yielding two control groups (Control-first and Control-second).

**Figure 3:**
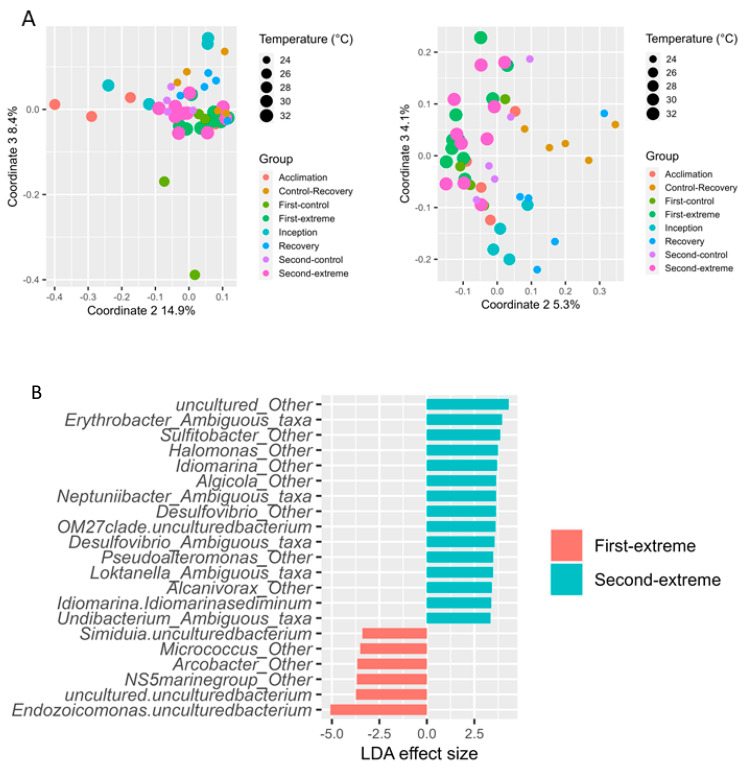
Effect of extremely high temperatures on gill microbiome. (**A**) PCoA of weighted/unweighted unifrac matrices. (**B**) Taxa differentially represented in the first-extreme (32 degrees) vs. second-extreme (33 degrees) treatment groups, detected with LEfSe.

Principal coordinates analysis (PCoA) (Figure 3A) indicated a distinct separation between the two experimental temperatures and the ambient groups. Accordingly, ANOSIM analysis revealed significant differences between the 32 °C/33 °C groups and all other groups pooled (ANOSIM *p* ≤ 0.034, R = 0.66; for all pair-wise ANOSIM comparisons, see Supplementary Table S1).

Notably, a distinct separation in microbial composition (ANOSIM *p* = 0.001, R = 0.43, N = 10; Supplementary Figure S3a) was also observed when directly comparing the first and second extreme temperature groups. The 1-degree temperature increase was sufficient to drive a significant increase in Shannon diversity (3.3 ± 0.8 vs. 4.1 ± 0.9, *p* = 0.01), resulting from compositional changes in 21 genera identified by LEfSe (Figure 3B, Supplementary Figure S3b). The majority (15) of these were overrepresented in the 33 °C group; these included the genus *Desulfovibrio*, one of the genera characterizing the “summer-like” group in the seasonal warming experiment. Conversely, only six genera were overrepresented in the 32 °C group; these included the genus *Endozoycomonas*, which was shown to decrease in the “summer-like” group of the seasonal warming experiment.

### 3.3. Effect of Extreme Cold on Oysters and Their Gill Microbiome

**Figure 4:**
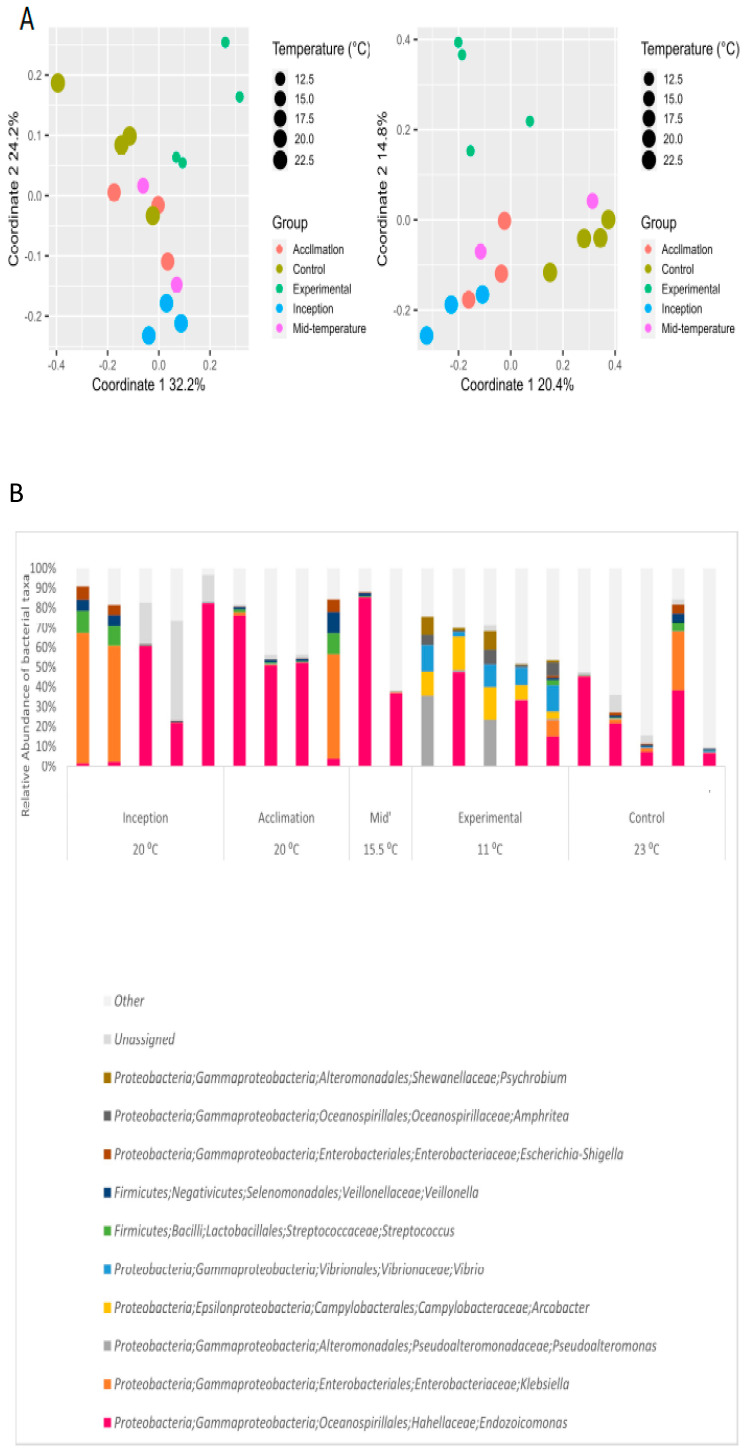
Effect of extremely low temperature on gill microbiome. (**A**) PCoA of weighted/unweighted unifrac matrices. (**B**) Relative abundances of dominant genera (appearing in at least two samples with a minimum RA of 5%).

Oysters were exposed to Western Mediterranean winter seawater temperatures typical to the coastal sites of Spain and Northern Italy [27,28] in order to determine the possible effects of this temperature on *S. spinosus* microbiota should its invasive range expand to include that region. Oysters were collected during winter (20 °C) and gradually exposed to 11 °C. In contrast to all previous experiments, all five oysters from the experimental group (11 °C) showed stress symptoms, manifested in keeping their valves closed most of the time, and two of them hardly responded to any external stimulus. The 12 control oysters remained normal-looking throughout the experiment. A divergence in the gill microbiome composition of the stressed oysters compared to the other groups was observed by PCoA (Figure 4A) and corroborated by PERMANOA (temperature effect: *p* = 0.001, R = 18%; group effect: *p* = 0.03, R = 28%).

Strikingly, the two stressed oysters from the experimental group did not retain any *Endozoicomonas*, whereas this genus was present in all other oysters throughout the experiment (Figure 4B). All the oysters under the extreme cold temperature featured certain distinct bacteria at relatively high abundance compared to the other groups, and the relative abundance of these taxa in the two stressed oysters was even higher. These included the genera *Arcobacter* and *Vibrio* (class γ-proteobacteria), the family *Colwelliaceae* (class γ-proteobacteria), and the genus *Pseudoalteromonas* (class γ-proteobacteria). These findings indicate that the oysters were stressed by the cold water temperature, leading some putatively beneficial members of the microbiome to be reduced to a below-detection level while genera known to include opportunistic pathogens of invertebrates, such as *Vibrio*, increased.

## 4. Discussion

### 4.1. Bacterial Gill Composition Is Strongly Affected by Temperature

In this study, we used the Lessepsian migrant *S. spinosus* oysters in order to examine the effect of exposure to a broad range of temperatures on the dynamics and composition of their gill microbiome, which may affect the future geographical expansion range of an invasive bivalve.

First, we examined how normal seasonal temperature fluctuations of the invaded habitat influence the gill microbiome. Notably, the temperature-shifted bacterial communities demonstrated substantial changes in composition in the warming experiment (Figure 2). The summer-like composition was more diverse than that observed at the ambient temperature and comprised several unique taxa. An intriguing example of this is the genus *Ruegeria*, which prospered in the summer-like group and then remained relatively abundant in the recovery (ambient winter temperature) group. Our results offer direct support for a previous study carried out under natural conditions, which revealed seasonal variations in the bacterial community of *S. spinosus* in the Mediterranean Sea [17]. It has been previously suggested that oyster-associated *Endozoicomonas* species are significantly affected by temperature changes and tend to thrive in their natural habitat during winter [17,29]. These observational studies targeting natural communities were supported by laboratory experiments conducted on isolated strains, which suggested the optimal in vitro growth temperature for *Endozoicomonas montiporae* is 25 °C [30], and for *Endozoicomonas elysicola*, it is between 25 and 30 °C, with growth stopping completely at 37 °C [31]. Accordingly, in the current warming experiment, the relative abundance of *Endozoicomonas* decreased markedly upon exposure to the peak summer temperature of 31 °C and returned to baseline levels after the recovery group had been returned to ambient conditions (18 °C, Figure 2), indicating the strain or strains associated with *S. spinosus* likely prefer the 25–30 °C range.

### 4.2. Oyster Symbionts in an Era of Climate Change

The average annual water temperature elevation rate between the years 1982 and 2012 was 0.035 ± 0.007 °C in the entire Mediterranean Sea and 0.05 °C in the Levantine Basin [32]. Currently, the annual water temperature along the Israeli coast fluctuates between 16 and 31 °C [17]. However, the Mediterranean Sea is predicted to warm up by the end of the 21st century by 0.5–2.6 °C, with the highest warming expected in its eastern basin [32]. It is often argued that ocean warming will result in an accelerated rate of marine invasions [33,34,35], which in turn will lead to the replacement and eradication of native species [36,37]. Understanding the effects of global warming on invasive species and their response to a continuous warming is thus highly important [33,34,35,37]. Our results indicate that a mere 2 °C shift in seawater temperature significantly alters the bacterial communities of oyster gills. As an example, the genus *Arcobacter*, which is commonly associated with bivalves [38] and considered a pathogen [39,40,41], was relatively prevalent in the two warmer temperature groups (Figure 3). Several studies indicated that elevated seawater temperature may lead to a change in the host bacterial communities [42] and may also lead to the emergence of pathogens or the activation of their virulence genes [43,44,45]. However, none of the oysters in the global warming experiments demonstrated any signs of physical deterioration and maintained normal functionality. Moreover, *Endozoicomonas*, while markedly affected by temperature elevation, did not disappear entirely, retaining a mean relative abundance of 17% (median 8%) and a minimal relative abundance of 2.5%. This genus is the dominant genus in the gills of these oysters and a core member of many other marine invertebrates, including sponges [46,47], corals [30,48,49], and other mollusks [15,31,50,51]. Previous studies have demonstrated that maintaining stable bacterial communities may assist the host in preventing disease, as has been suggested for the corals *Montipora aequituberculata* [52] and *Porites astreoides* [53]. Therefore, we expect that *S. spinosus* oysters will survive further global warming, perhaps at the expense of more heat-sensitive oyster species.

### 4.3. Oysters and Endozoicomonas Bacteria Are Strongly Impacted by Cold Temperatures Prevailing in the Western Mediterranean Sea

As a response to climate change, organisms are predicted to shift their geographical ranges toward colder environments [54]. As water temperatures increase, it is expected that the cooler Western Mediterranean Sea will become increasingly susceptible to species-range expansion. Most of the invasive thermophilic species in the Eastern Mediterranean Sea are of Red Sea origin and gradually expand their distribution westward [55]. We were, therefore, intrigued by the question of whether *S. spinosus* would be able to follow such a pattern of geographic expansion.

In the extreme cold experiment, several oysters displayed a decline in health and in the relative abundance of *Endozoicomonas*, with a concomitant increase in the relative abundance of several other taxa, including *Vibrio* and *Pseudoalteromonas.* It is likely that a decline in the relative abundance of *Endozoicomonas* may provide an opportunity for pathogens to colonize the host [56,57]. Alternatively, such a shift in the bacterial community could represent an overall physiological deterioration in the oyster’s health due to exposure to colder temperatures. Indeed, in contrast to warmer temperature exposures, a noticeable health deterioration occurred when oysters were exposed to 11 °C and exhibited symptoms of stress or mortality, accompanied by the emergence of distinct bacterial taxa, which could be considered opportunistic pathogens, including *Arcobacter* [41], *Vibrio* [44], *Colwelliaceae* [58], and *Pseudoalteromonas* [59]. The severely affected oysters had an even higher relative abundance of these genera while not retaining any detectable *Endozoicomonas*. This finding suggests that the oyster host is more susceptible to the cold temperature regime than its *Endozoicomonas* symbiont, as has been previously noted in corals [56,57], and that the current winter temperatures of the Western Mediterranean Sea might limit the geographical expansion of *Spondylus* oysters, at least in the near future.

## 5. Conclusions

This study indicates that seawater warming will allow the marine invasive oyster *S. spinusus* to retain its current dominance in the Eastern Mediterranean while colonizing coastal areas further northwest of its current geographic range. However, as long as the winter water temperatures in the northwesterly regions of the Mediterranean remain as low as 11 °C, these regions are expected to remain uncolonized by *S. spinosus* and possibly by other invasive Red Sea oysters, such as *Chama pacifica* [60]. The results imply that the microbial symbionts can be dramatically affected by temperature shifts, which, in turn, could affect the invasion expansion of the invasive species that host them, either by promoting bacterial pathogenesis or by negatively impacting mutualists. This study provides an experimental framework to examine an invasive holobiont, including the dynamics of its microbial symbionts. Similar studies on other invasive species will allow us to identify environmental predictors relevant to invasion trajectories in the marine environment and may also improve management practices in the Mediterranean Sea and beyond.

## Figures and Tables

**Table 1:** (**A**) Experiments inducing shifted temperature regimes on the invasive oyster *Spondylus spinosus.* (**B**) Detailed temperature shifts and sampling groups per experiment.

A.									
Experiment	Warming Experiment			Global Warming Experiment			Extreme Cold Experiment		
Inception date	4 Feburary 2016			5 October 2016			5 April 2016		
Number of oysters	33			49			22		
Onset temperature (°C)	17			29			20		
Target temperature (°C)	31			32 & 33			11		
**B.**									
**Group**	**Days since Inception**	**Temp** **erature** **(°C)**	**Sample Size**	**Days since Inception**	**Temp** **erature** **(°C)**	**Sample Size**	**Days since Inception**	**Temp** **erature** **(°C)**	**Sample Size**
Inception	0	17	5	0	29	5	0	20	5
Acclimation	10	18	5	8	27	4	7	20	4
Mid-temperature	13	24	3	-	-	-	9	15	2
Experimental	28	31	5	23	32	10	23	11	5
				36	33	10			
Control	28	18	4	23	26	5	23	23	5
				36	24	5			
Recovery	47	18	5	53	24	5	-	-	-
Control- recovery	47	18	5	53	24	5	-	-	-

## Data Availability

Raw 16S data and metadata have been deposited in NCBI’s Sequence Read Archive (SRA), Project ID PRJNA1060268.

## Supplementary Materials

The following supporting information can be downloaded at https://www.mdpi.com/article/10.3390/microorganisms12010197/s1. Supplementary Figure S1: Relative abundance patterns of taxa characterizing the summer-like group in the seasonal warming experiment; Supplementary Figure S2: Taxa differentially distributed between summer-like group (green) and all other oyster groups (red) in the warming experiment.; Supplementary Figure S3: Microbiome difference between two high temperature extremes (32C and 33C).; Supplementary Table S1: Unweighted UniFrac-based one-way ANOSIM (*p*-value and R-value) between gills bacterial communities of *Spondylus spinosus* from global warming experiment.
