# Supplementary material for: Host–Microbiome Interactions in a Changing Sea: The Gill Microbiome of an Invasive Oyster under Drastic Temperature Changes
Source: Microorganisms. 2024 Jan 18;12(1):197. doi: 10.3390/microorganisms12010197 (PMC10819450; doi:10.3390/microorganisms12010197)

Supplementary Data

Figure S1. Relative abundance patterns of taxa characterizing the summer-like group in the seasonal warming experiment..

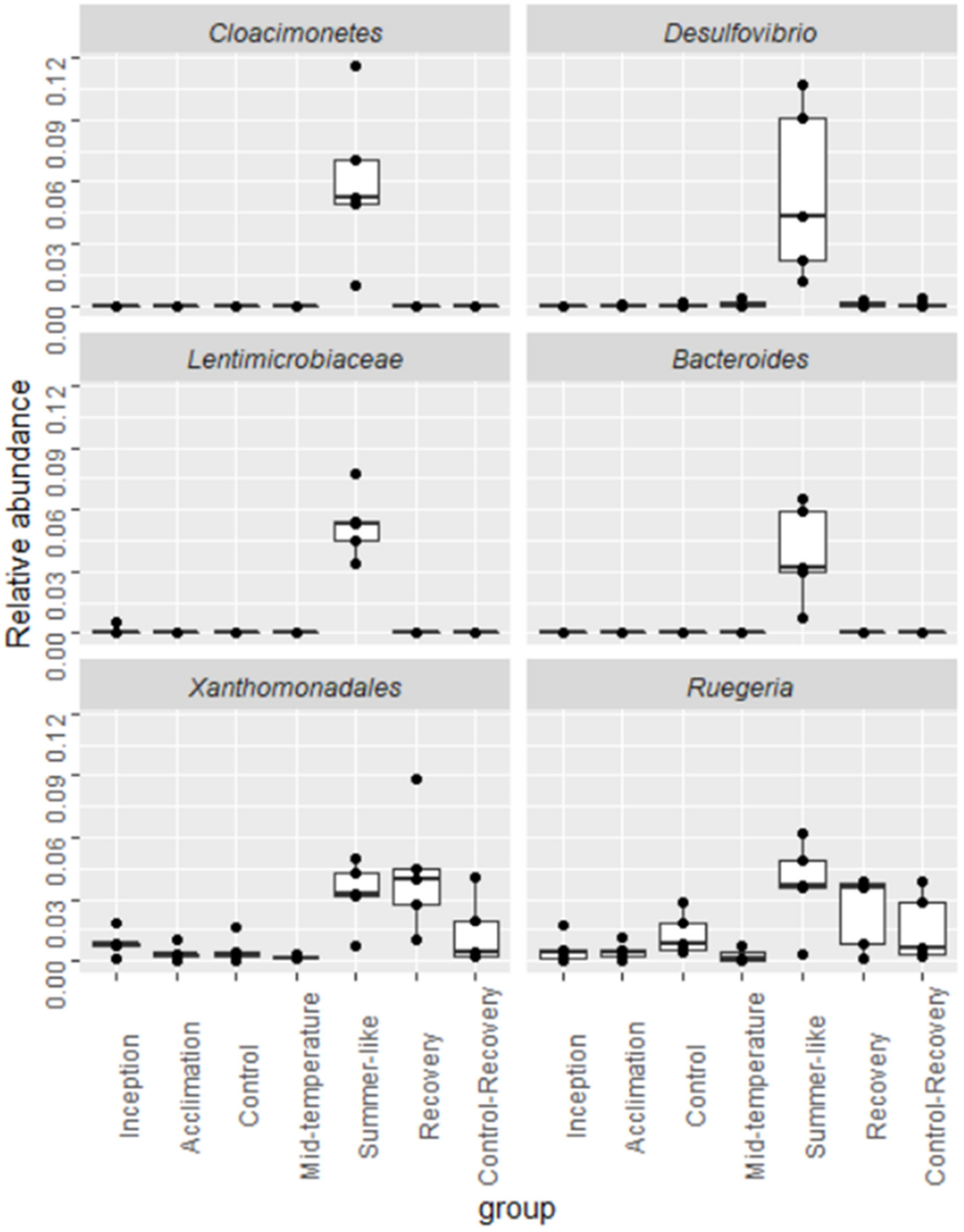

Figure S2.Taxa differentially distributed between summer-like group (green) and other oysters (red) warming experiment. LEfSe algorithm was used with default settings (p KW=0.05; LDA threshold 3)

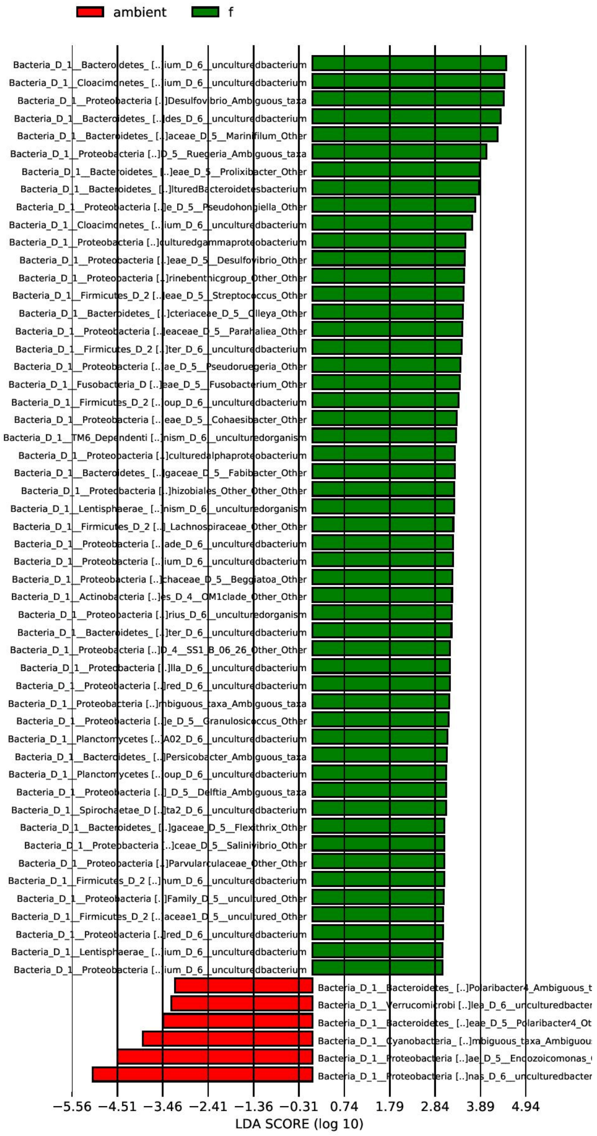

Table S1. Unweighted UniFrac-based One way ANOSIM (P-value and R- value) between gills bacterial communities of *Spondylus spinosus* from Global-warming experiment. *Bonferroni adjusted p- values < 0.05.

P-value	Inception	Acclimation	1st experimental	1st control	2nd experimental	2nd control	Recovery	Control-recovery
Inception	0							
Acclimation	0.7728	0						
1st experimental	0.0196	0.0336	0					
1st control	0.2296	1	0.1372	0				
2nd experimental	0.0336	0.0364	0.0056	0.0168	0			
2nd control	0.4676	1	0.07	1	0.056	0		
Recovery	0.2604	0.2128	0.0168	0.2156	0.0168	0.9128	0	
Control-recovery	0.2604	1	0.0084	1	0.0028	1	1	0

R-value	Inception	Acclimation	1st experimental	1st control	2nd experimental	2nd control	Recovery	Control-recovery
Inception	0							
Acclimation	0.6563	0						
1st experimental	0.9843	0.6598	0					
1st control	0.8188	0.08125	0.3825	0				
2nd experimental	0.901	0.5745	0.4002	0.592	0			
2nd control	0.6375	0.09375	0.5731	0.056	0.4633	0		
Recovery	0.9875	0.55	0.7338	0.64	0.4204	0.472	0	
Control-recovery	0.6563	0.1125	0.6007	0.192	0.6065	0.028	0.156	0

Figure S3. Microbiome difference between two high temperature extremes (32C and 33C). **A.**PCoA of weighted UniFrac distances; B: Boxplots of 21 genera discriminating between the two extreme temperatures (identified by Lefse, Fig. 3b).

(A)

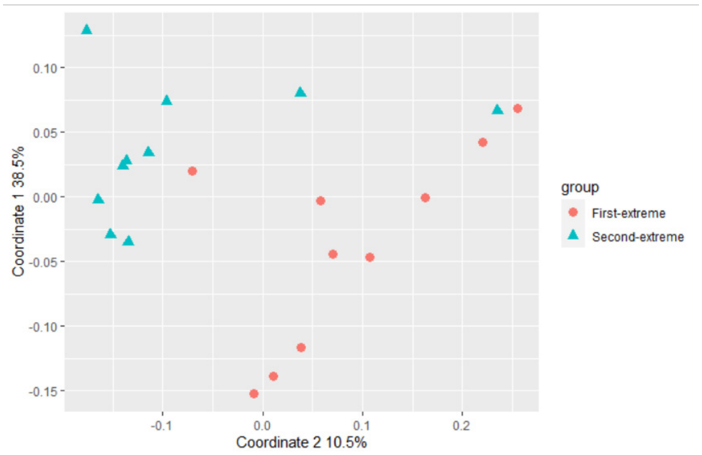

(B)

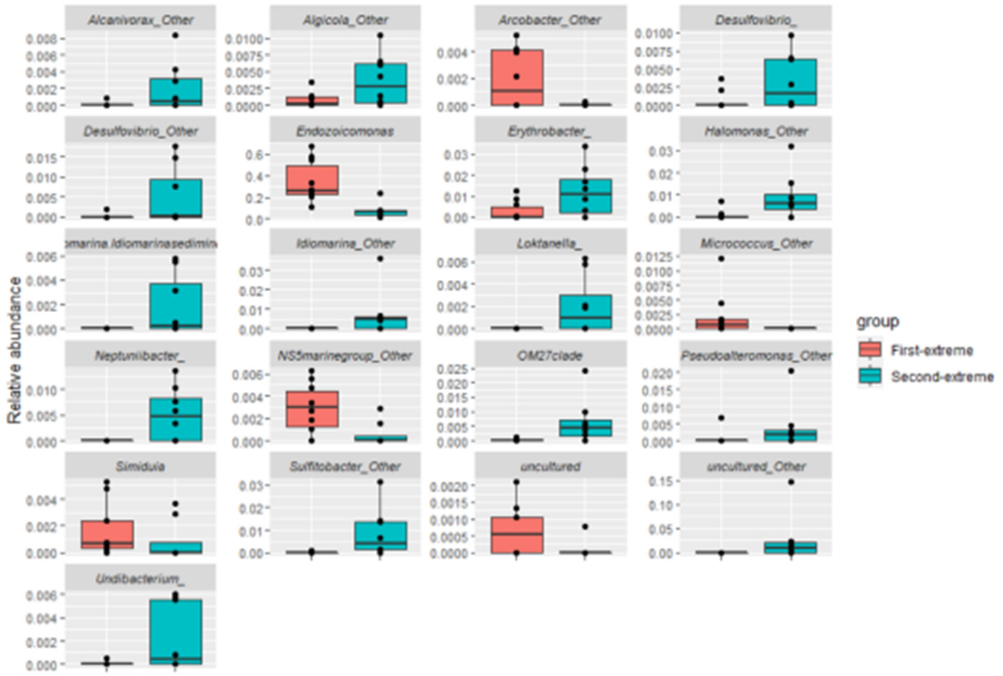

Supplement: Supplementary file 1 [file microorganisms-12-00197-s001.zip › microorganisms-2781747-supplementary.pdf]
